# miR-146b promotes cell proliferation and increases chemosensitivity, but attenuates cell migration and invasion via FBXL10 in ovarian cancer

**DOI:** 10.1038/s41419-018-1093-9

**Published:** 2018-11-08

**Authors:** Meina Yan, Xinxin Yang, Rong Shen, Chengjiang Wu, Hui Wang, Qing Ye, Peifang Yang, Lubin Zhang, Miao Chen, Bing Wan, Qinqin Zhang, Sheng Xia, Xiaodong Lu, Genbao Shao, Xiaoming Zhou, Jun Yu, Qixiang Shao

**Affiliations:** 1 Department of Immunology, Key Laboratory of Medical Science and Laboratory Medicine, School of Medicine, Jiangsu University, Zhenjiang, 212013 Jiangsu China; 2 Department of Pathology, Nanjing Drum Tower Hospital, The Affiliated Hospital of Nanjing University Medical School, Nanjing, 210008 Jiangsu China; 3 Department of Gynecology and Obstetrics, Affiliated Hospital of Jiangsu University, Zhenjiang, 212001 Jiangsu China; 4 Department of Pathology, Affiliated People’s Hospital of Jiangsu University, Zhenjiang, 212001 Jiangsu China; 5 Department of ICU, Affiliated Hospital of Jiangsu University, Zhenjiang, 212001 Jiangsu China; 6 Department of Wuxi Maternal and Child Health Care Hospital, Wuxi, 214000 Jiangsu China

## Abstract

Epithelial ovarian carcinoma (EOC) is the most lethal gynecologic malignancy. However, the molecular mechanisms remain unclear. In this study, we found that miR-146b was downregulated in EOC and its expression level was negatively correlated with the pathological staging. Follow-up functional experiments illustrated that overexpression of miR-146b significantly inhibited cell migration and invasion, and increased cell proliferation, but it also improved the response to chemotherapeutic agents. Mechanistically, we demonstrated that miR-146b exerted its function mainly through inhibiting F-box and leucine-rich repeat protein 10 (FBXL10), and upregulated the Cyclin D1, vimentin (VIM), and zona-occludens-1 (ZO-1) expression in EOC. These findings indicate that miR-146b–FBXL10 axis is an important epigenetic regulation pathway in EOC. Low miR-146b may contribute to cancer progression from primary stage to advanced stage, and may be the promising therapeutic target of EOC.

## Introduction

Of all gynecologic malignancies, ovarian cancer is the most lethal gynecologic malignancy^1,2^. More than 85% of the instances of human ovarian cancer are epithelial ovarian carcinoma (EOC)^3^. Despite recent advances in molecularly targeted therapy and immunotherapy such as anti-PD-1/PD-L1 antibody and CAR-T therapy, the 5-year survival rate of advanced EOC patients falls below 25%^4,5^. This is primarily because EOC has few early or specific symptoms, and two-thirds of patients had advanced-stage and high-grade cancer at the time of diagnosis. In addition, ovarian cancer can spread by direct invasion to adjacent organs or by transcoelomic metastasis through ascites^6^. However, the molecular mechanisms of EOC tumorigenesis and metastasis are still not completely understood.

MicroRNAs (miRNAs) are short noncoding RNAs that regulate gene expression by binding the 3′-untranslated regions (UTR) of mRNAs, inducing direct mRNA degradation, or translation inhibition^7^. Accumulating data have shown that miRNAs are associated with EOC initiation, progression, and metastasis^8–11^. There has been some reports of miR-146b in other cancers^12,13^. The microRNA microarrays indicated that miR-146b was a differentially expressed miRNA in ovarian cancer^14^; however, the functional role of miR-146b in EOC has rarely been investigated.

The F-box and leucine-rich repeat protein 10 (*FBXL10*, also known as *KDM2B* or *Jhdm1b*) belongs to the JmjC domain-containing histone demethylases, and controls stem cell self-renewal, cell senescence, and tumorigenesis^15,16^. As an important tumor accelerator, FBXL10 has been reported in pancreatic cancer, leukemia, and breast cancer^17–19^. However, whether FBXL10 modulates EOC development remains largely unclear.

In the present study, we found that miR-146b inhibited the expression of FBXL10 and might play a dominant role in EOC progression. MiR-146b downregulated the FBXL10 expression, upregulated the zona-occludens-1 (ZO-1) expression, and led to cell invasion suppression, but it also enhanced the Cyclin D1 expression, promoted cell proliferation, and increased the chemosensitivity. Our findings may imply that in the early stage of EOC, the high expression of miR-146b contributes to the maintenance of desmosomes for the intercellular connection, and promotes the growth of carcinoma locally. Subsequently, at the later stage of EOC, decreasing miR-146b promotes the FBXL10 upregulation, which promotes tumor metastasis and chemoresistance. Thus, the miR-146b–FBXL10 axis might be the check point of the EOC therapy.

## Results

### MiR-146b decreased in EOC tissues and correlated with EOC progression

**Fig. 1:**
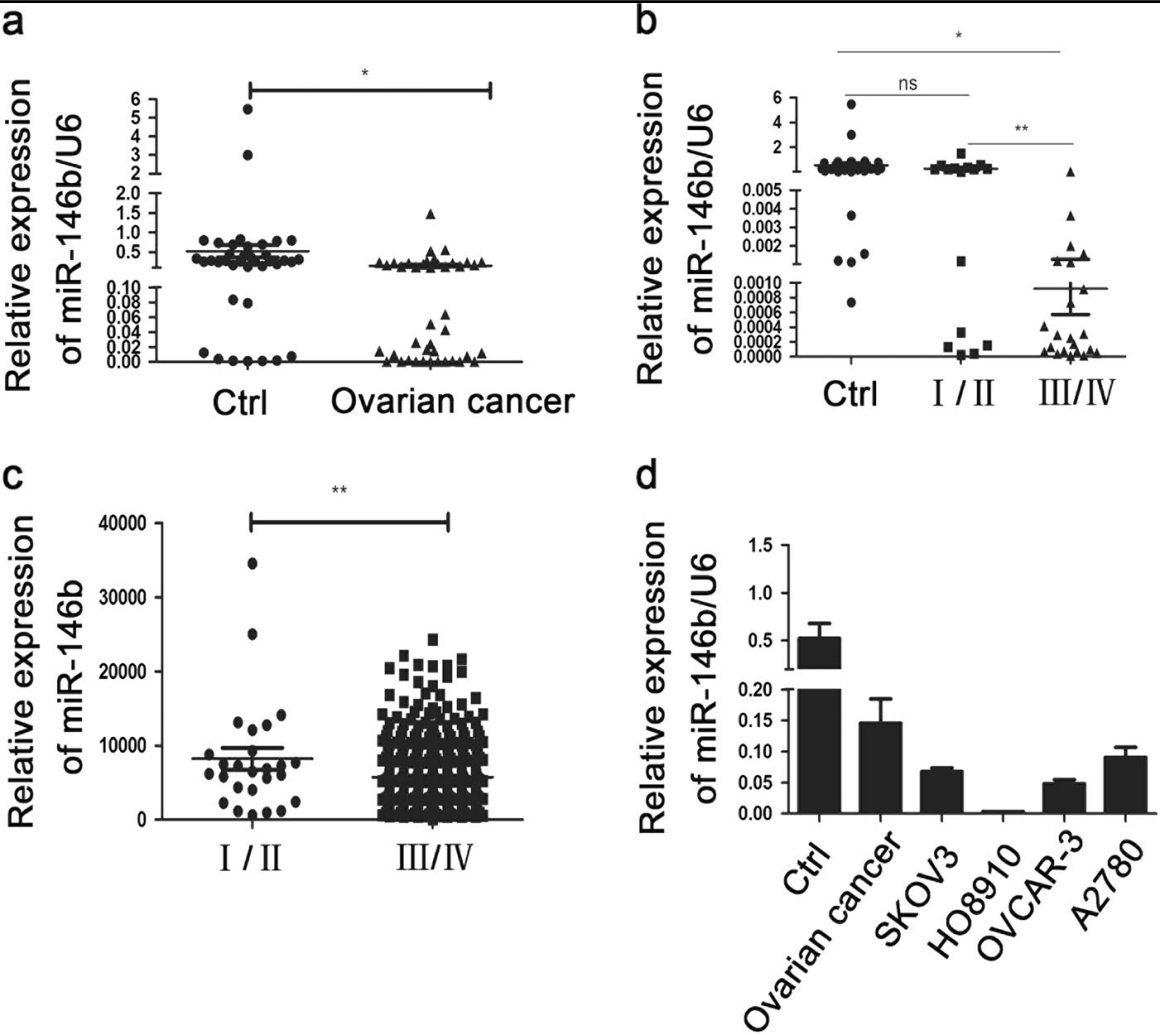
MiR-146b was downregulated in human ovarian cancer samples and cell lines. **a** qPCR analysis of miR-146b expression in ovarian tumors (*n* = 42) and control samples (*n* = 38). **b** qPCR analysis of miR-146b expression in ovarian tumors with different malignant grades (I/II stage, *n* = 16, III/IV stage *n* = 22). **c** miR-seq analysis of miR-146b expression from the TCGA database in different malignant grades (I/II stage, *n* = 22, III/IV stage, *n* = 443). **d** The relative expression level of miR-146b in four ovarian cancer cell lines. Data are presented as means ± SD from three individual experiments. *p < 0.05, **p < 0.01, ns not significant

To identify the role of miR-146b in EOC, we first analyzed the expression levels of miR-146b in EOC samples through stem-loop quantitative PCR (qPCR). Clinical features of this study are summarized in Table 1. As depicted in Fig. 1a, miR-146b was downregulated in human EOC samples (*P* < 0.05), compared with the control samples. Furthermore, the expression level of miR-146b in later grade III/IV stage ovarian cancer samples was significantly decreased compared with the grade I/II samples (Fig. 1b). Moreover, microRNA sequencing (miRNA-seq) analysis from the Cancer Genome Atlas database indicated that the expression level of miR-146b was also downregulated in the stage III/IV samples compared with stage I/II samples (Fig. 1c). Finally, we found that miR-146b was lower in EOC cell lines compared with the control samples (Fig. 1d). Thus, these results imply that miR-146b may play a role in the progression of EOC.

**Table 1:** The general and clinical information of the tissue specimens in this study

Clinic pathological features	Ctrl ovary, *n* = 38	Ovarian cancer, *n* = 42
Age, mean ± SD (range)	–	47 ± 14
Clinical stage		
I	0	2
II	0	14
III	0	12
IV	0	10
Unknown	0	4
Histotype		
Papillary-serous	0	19
Mucinous	0	3
Endometrioid	0	2
Clear cell	0	1
Unknown	0	17

### MiR-146b overexpression changes the tumor cells phenotype from an invasive phenotype to a proliferative state

**Fig. 2:**
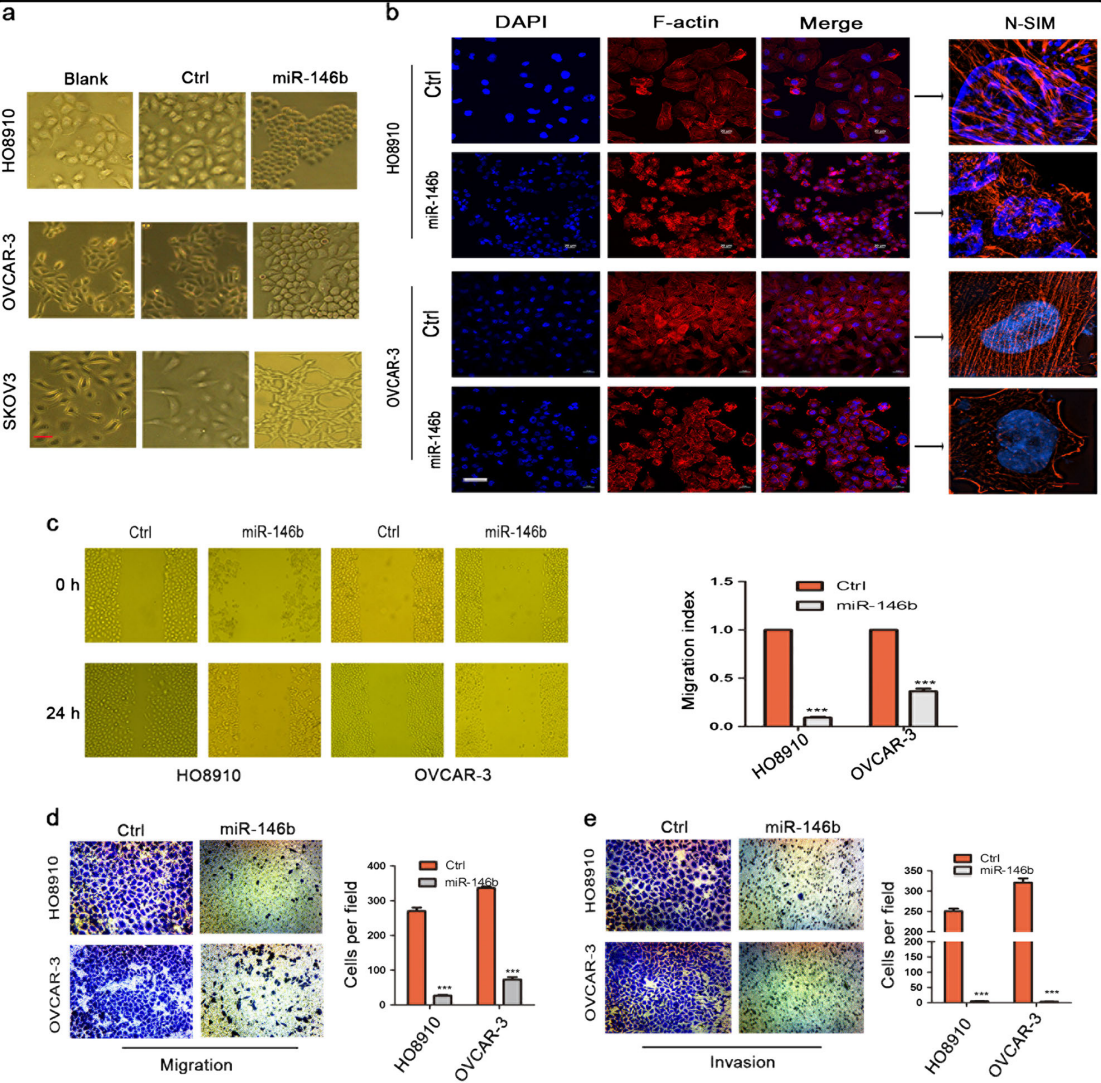
MiR-146b inhibited the migration and invasion of ovarian cancer cells. **a** Changes in cell morphology of ovarian cancer cells after miR-146b overexpression. Scale bars represent 50 μm. **b** Representative images of F-actin staining of ovarian cancer cells with miR-146b overexpression; nuclei were stained with DAPI. Scale bars represent 40 µm. **c** Wound healing assay of HO8910 and OVCAR-3 cells with miR-146b overexpression. Representative pictures are shown at 0 and 24 h after the wound was made. **d**, **e** In vitro transwell migration (200×) (**d**) and Matrigel invasion assay (200×) (**e**) of HO8910 and OVCAR-3 cells after miR-146b overexpression. ***p < 0.001

To further explore the functional role of miR-146b in EOC, ovarian cancer cell lines with miR-146b overexpression were established by lentivirus infection (Figure S1a, b). Morphologically, the ovarian cancer cells with miR-146b overexpression appeared to be smaller and enhanced the intercellular connection compared with the control cells (Fig. 2a and Figure S1c). Previous studies have demonstrated that miR-146b could induce cell apoptosis via TRAF6 and IRAK1^20^; thus, we investigated whether miR-146b overexpression in ovarian cancer cells also caused apoptosis. As depicted in Figure S1d, we demonstrated that miR-146b did not downregulate TRAF6 nor IRAK1. Furthermore, we have not seen an increased in apoptosis of cells after miR-146b overexpression (Figure S1e). To further explore the potential function of miR-146b in ovarian cancer, we observed the cytoskeletal arrangement with miR-146b overexpression. As presented in Fig. 2b, miR-146b injured cell cytoskeleton, and the arrangement of F-actin was disordered, folded, and damaged. We further discovered that miR-146b inhibited cell migration, as indicated by the wound healing assay (Fig. 2c). Similarly, the transwell assay revealed that enforced miR-146b expression significantly inhibited cell migration and invasion (Fig. 2d, e).

**Fig. 3:**
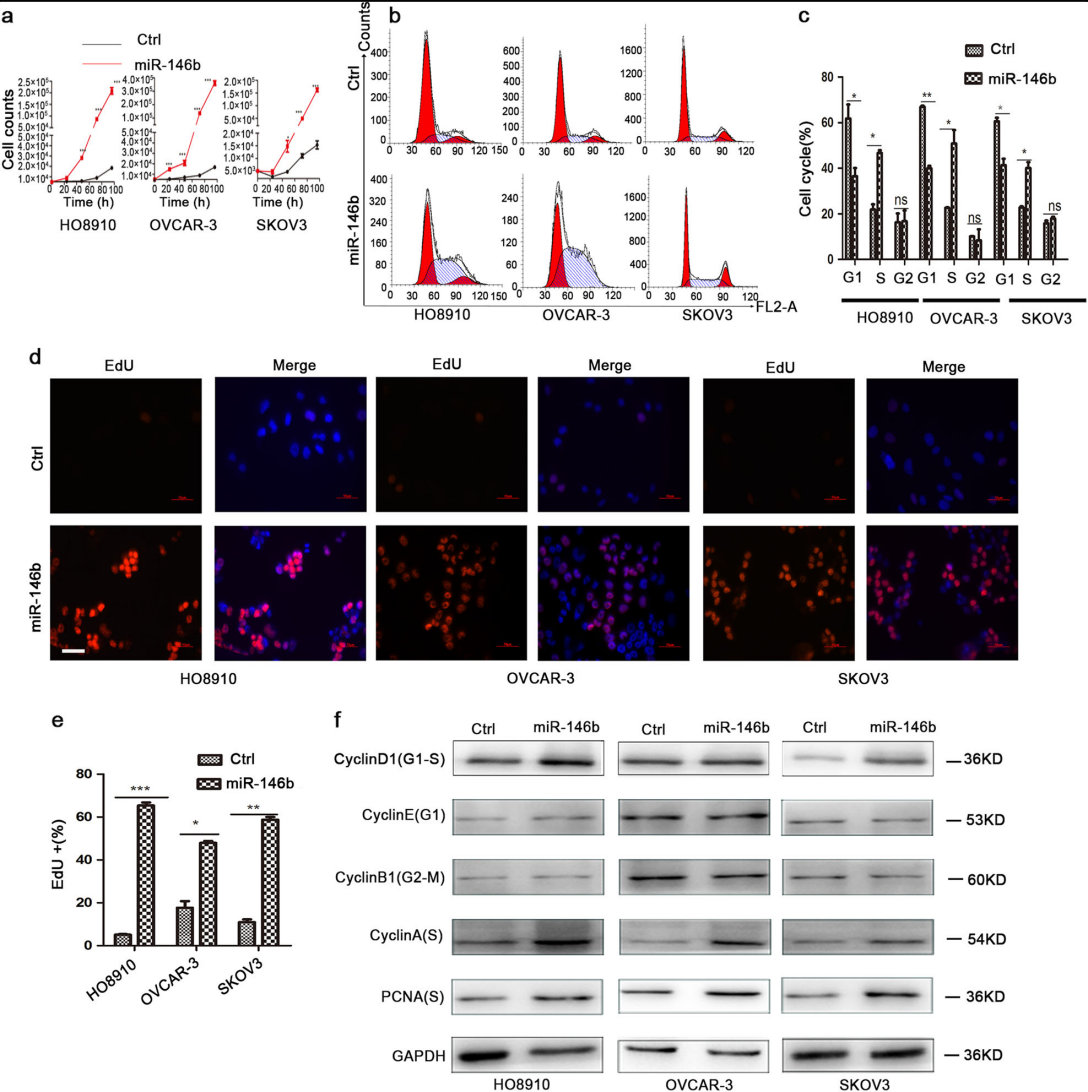
MiR-146b overexpression promoted ovarian cancer cell proliferation. **a** Effects of miR-146b on cell proliferation in HO8910, OVCAR-3, and SKOV3 cells were analyzed using the cell count assay. **b** Flow cytometry analysis of cell cycle distribution of ovarian cancer cells transduced with miR-146b lentrivirus. **c** Quantification of the percentage of cell cycle is shown. **d** EdU assay was used to evaluate the proliferation of miR-146b-overexpressing cells. Representative images for EdU-positive ovarian cancer cells (red) and Hoechst-stained nuclei (blue) are shown. Scale bars represent 50 μm. **e** Quantification of the percentage of EdU-positive cells is shown. **f** Detection of Cyclin D1, Cyclin E, Cyclin B1, Cyclin A, and PCNA protein expression of ovarian cancer cells by western blot analysis. *p < 0.05, **p < 0.01, and ***p < 0.001; ns not significant

Cell proliferation was another hallmark of cancer. Our results demonstrated that the number of cells with miR-146b overexpression greatly increased compared with the control cells (Figure S2). Cell count assay revealed that miR-146b overexpression significantly boosted cell proliferation (Fig. 3a). Flow cytometry (FCM) analyses further showed that miR-146b overexpression increased the percentage of S-stage cells (Fig. 3b, c). The 5-ethynyl-2′-deoxyuridine (EdU) assay also indicated that the percentage of EdU-positive cells was significantly higher in cells with miR-146b overexpression (Fig. 3d, e). A western blot analysis revealed that miR-146b significantly upregulated the expression level of Cyclin D1, Cyclin A, and Proliferating cell nuclear antigen (PCNA) (Fig. 3f). The above results suggest that miR-146b reduced cell migration and invasion, but enhanced proliferation. It acts like a switch between invasive and proliferative states.

### MiR-146b enhanced EOC cell chemosensitivity

**Fig. 4:**
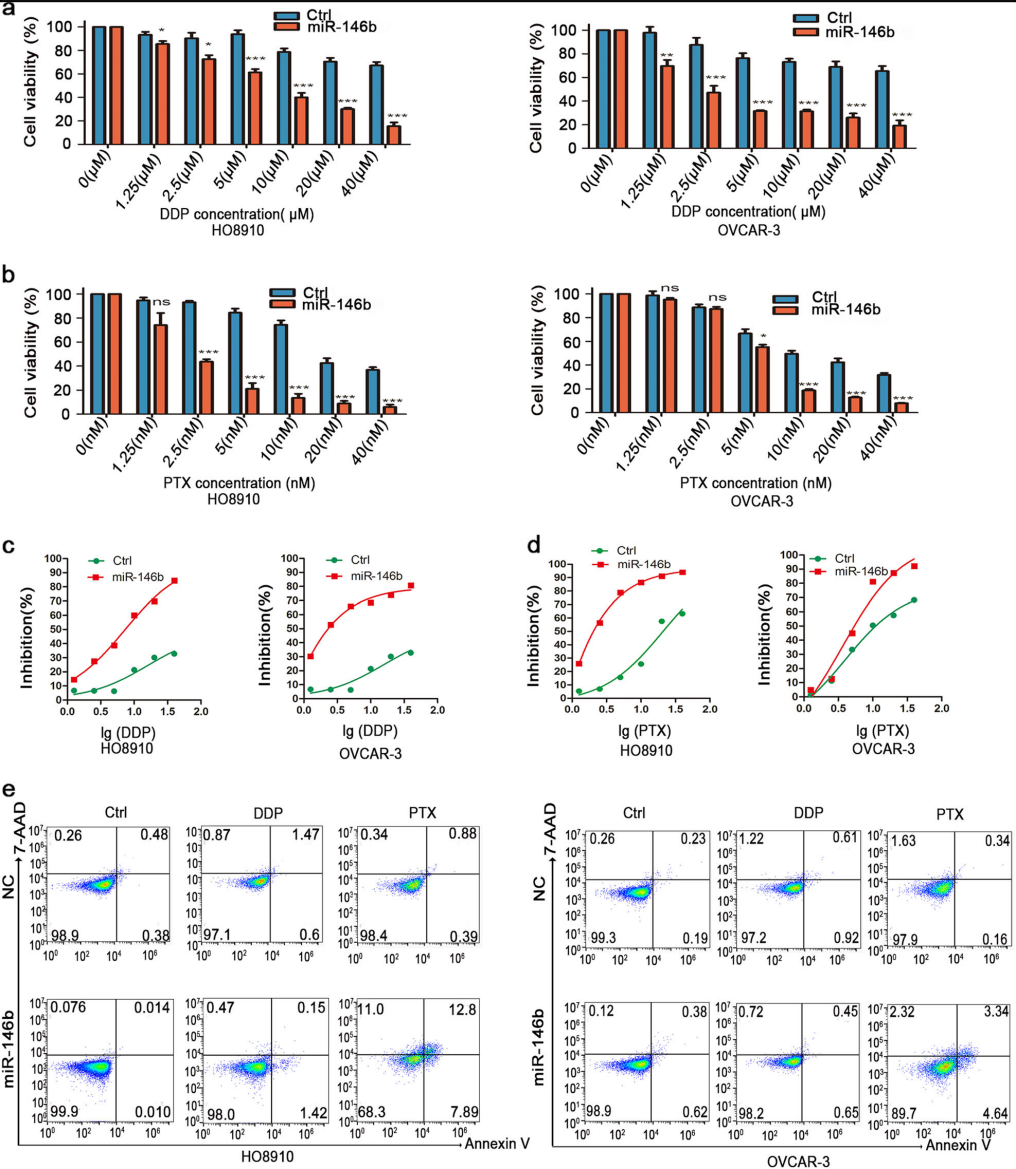
miR-146b enhanced ovarian cancer cell sensitivity to chemotherapeutic reagents. **a**, **b** Histograms showing the percentage of cellular proliferation inhibition rate of ovarian cancer cells with different concentrations of DDP (**a**) and PTX (**b**). **c** Diagram line about different concentrations of DDP and the IC50 difference between Ctrl and miR-146b-overexpressing cells. **d** Diagram line about different concentrations of PTX and the IC50 difference between Ctrl and miR-146b-overexpressing cells. **e** Representative flow cytometry of apoptosis in HO8910 and OVCAR-3 ovarian cancer cells. Cells were treated with DDP (40 μM), PTX (10 nM) for 48 h, and then were stained with Annexin V and 7-AAD before subjecting to FCM for cell apoptosis analysis. *p < 0.05, **p < 0.01, and ***p < 0.001; ns not signifcant

A compelling piece of evidence is that most high-grade ovarian cancers often exhibit resistance to chemotherapy. The most successful anti-cancer drugs of EOC were cisplatin (DDP) and paclitaxel (PTX), which target the rapidly dividing cells. MiR-146b overexpression promoted cell cycle progression, suggesting that it might act on cell’s response to chemotherapeutic drugs. To examine whether miR-146b overexpression induced specific changes of chemosensitivity, cells with overexpressing miR-146b were treated with DDP and PTX. Overexpression of miR-146b induced growth inhibition after treatment with DDP and PTX (Fig. 4a, b). The results further indicated that miR-146b decreased the half-maximal inhibitory concentration (IC50) of DDP and PTX (Fig. 4c, d). Moreover, the Annexin V-APC/7-AAD double-staining assessment revealed that cells with miR-146b overexpression markedly promoted cell apoptosis after PTX treatment (Fig. 4e). These data suggest that an increase in proliferation is crucial for high response to chemotherapeutic agents, and reduced proliferation may increase cell chemoresistance.

### MiR-146b directly targeted FBXL10 in ovarian cancer

**Fig. 5:**
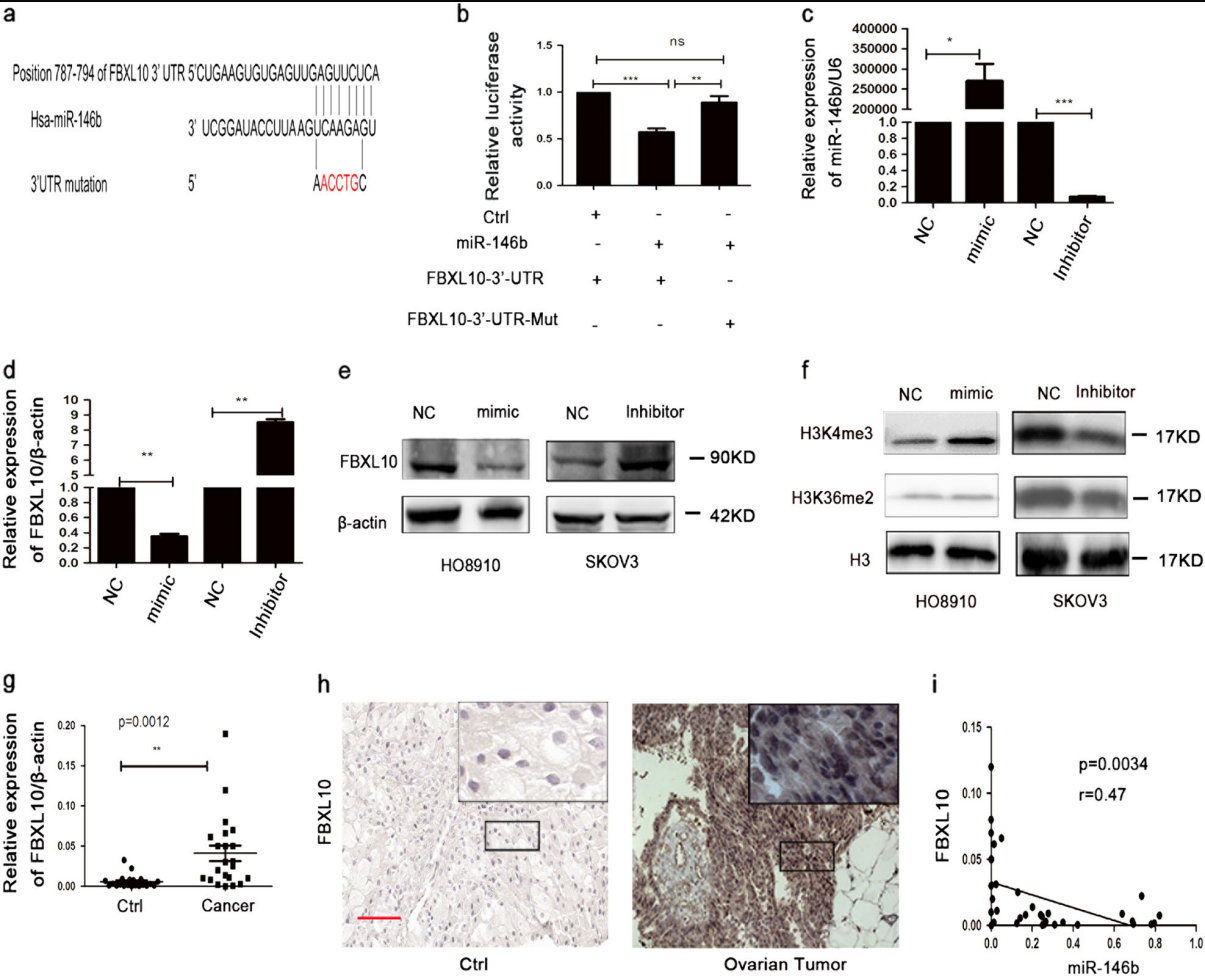
MiR-146b directly targeted FBXL10. **a** Schematic representation of the miR-146b and its targeting sites in the 3′-UTR of *FBXL10*, indicating the binding sites and the corresponding mutations. **b** miR-146b directly interacted with the *FBXL10*-3′-UTR. The 293T cells were co-transfected with the luciferase reporter vector, and pcDNA3.1-pri-miR-146b or control plasmids into 293T cells. The luciferase activity was measured after 24 h according to the protocol described in Materials and methods section. **c** Levels of mature miR-146b were detected using qPCR after transfection with miR-146b mimics or miR-146b inhibitors for 24 h. **d**, **e** mRNA (**d**) and protein (**e**) levels of FBXL10 in HO8910 and SKOV3 cells transfected with miR-146b mimics or miR-146b inhibitors. **f** Relative levels of H3K4me3 and H3K36me2 transfected with miR-146b mimics or miR-146b inhibitors. **g**, **h** The expression level of *FBXL10* in ovarian cancer samples using qPCR (**g**) and immunohistochemical staining (**h**) (control samples, *n* = 21; cancer samples, *n* = 22). **i** Regression and correlation analysis between miR-146b and *FBXL10* expression in ovarian cancers (*n* = 37). Scale bars represent 100 μm. *p < 0.05, **p < 0.01, and ***p < 0.001; ns not significant

To explore the molecular mechanisms of miR-146b in EOC, we identified genes that were directly regulated by miR-146b. Through TargetScan 5.1 combined with PicTar and miRanda analyses, we found that the 3′-UTR of *FBXL10*, *ELAVL1*, and *Lin28a* genes exhibited a highly conserved seed sequence for the miR-146b (Fig. 5a and Figure S3a). Dual luciferase reporter assay further confirmed that miR-146b overexpression was capable of decreasing the luciferase activity of wild-type construct of *FBXL10*-3′-UTR. In contrast, the mutation of the *FBXL10*-3′-UTR clearly abrogated the inhibition of the luciferase activity caused by miR-146b overexpression. These findings indicated that miR-146b directly targets *FBXL10*, rather than *ELAVL1* and *Lin28a* (Figure S3b). Next, HO8910 and SKOV3 cells were transfected with miR-146b mimics or miR-146b inhibitors depending on the level of miR-146b (Fig. 5c). Further studies indicated that miR-146b overexpression or knockdown markedly changed the mRNA levels and protein expression levels of FBXL10 (Fig. 5d, e). The transwell assay further confirmed that miR-146b negatively regulated cell migration (Figure S3c). Previous studies have indicated that FBXL10 was a histone lysine demethylase that could target H3K4me3 or H3K36me2 for demethylation^15,21^; our results revealed that FBXL10 especially removed methyl groups from H3K4me3 in ovarian cancer cells (Fig. 5f). We finally investigated the expression of FBXL10 in EOC samples using qPCR and immunohistochemistry (IHC) assay. The results indicated that FBXL10 was significantly upregulated in EOC samples compared with control samples (Fig. 5g, h). The expression of *FBXL10* also had a negative correlation with miR-146b expression in these samples (Fig. 5i).

**Fig. 6:**
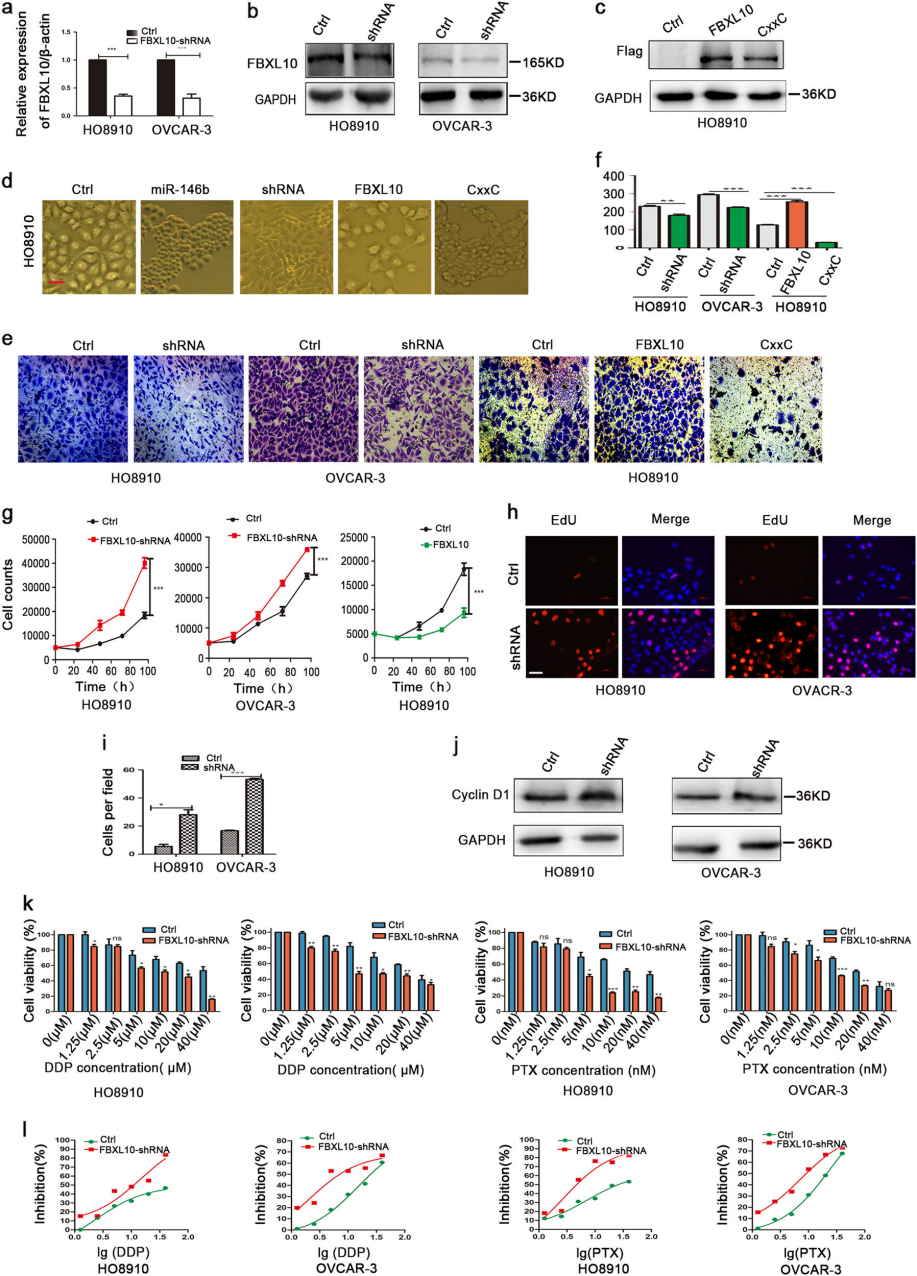
Functional analysis of FBXL10 in ovarian cancer. **a**, **b** shRNA efficiency analysis in ovarian cancer cells by qPCR (**a**) and western blot (**b**). **c** Wild-type FBXL10-Flag overexpression and FBXL10-CxxC(dele)-Flag overexpression in HO8910 cells. Immunostaining was performed with Flag antibody. **d** Representative images of ovarian cancer cells transduced with FBXL10-shRNA or FBXL10 lentivirus. Scale bars represent 50 μm. **e** The representative images of the transwell migration assay, respectively (200×). **f** Quantification of the transmembrane migration abilities of the ovarian cancer cells. **g** The effects of FBXL10 on cell proliferation using cell count assay. **h** EdU assay was used to evaluate the proliferation of Ctrl and shRNA of FBXL10 cells. Representative images for EdU-positive ovarian cancer cells (red) and Hoechst-stained nuclei (blue) are shown. Scale bars represent 50 μm. **i** Quantification of the percentage of EdU-positive cells is shown. **j** Detection of Cyclin D1 protein expression of ovarian cancer cells after FBXL10 knockdown by western blot analysis. **k** Histograms showing the percentage of cellular proliferation inhibition rate of ovarian cancer cells with DDP and PTX treatment after FBXL10 knockdown. **l** The IC50 difference of DDP/PTX between Ctrl and FBXL10-knockdown cells. *p < 0.05, **p < 0.01, and ***p < 0.001; ns not significant

To determine whether the function of miR-146b in EOC was mediated through *FBXL10*, we designed a short hairpin RNA (shRNA) that could target* FBXL10*, and the knockdown efficiency was verified through qPCR and western blot (Fig. 6a, b). In addition, we generated an HO8910 cell model that stably overexpressed FBXL10 or FBXL10-CxxC (CxxC domain deletion) (Fig. 6c). The CxxC zing-finger domain recognizes unmethylated CpG islands and recruits polycomb-repressive complex 1 to target genes^16^. First, we discovered that cells transduced with FBXL10-shRNA and FBXL10-CxxC domain deletion had closely connected clones, but cells with the FBXL10 overexpression appeared to exhibit a looser and more dispersed phenotype (Fig. 6d). The transwell assay demonstrated that both FBXL10 knockdown and FBXL10-CxxC deletion inhibited cell migration, while FBXL10 overexpression increased migration (Fig. 6e, f). The cell count assay and EdU assay indicated that FBXL10 knockdown increased cell proliferation; however, FBXL10 overexpression inhibited cell growth (Fig. 6g–i). Western blot analysis indicated that FBXL10 knockdown upregulated the expression of Cyclin D1 (Fig. 6j). FBXL10 knockdown also improved the response to DDP/PTX treatment (Fig. 6k, l). From these results, we can surmise that the role of miR-146b in regulating EOC is FBXL10-dependent in vitro, and FBXL10-mediated high migration is via its CxxC domain.

### MiR-146b enhanced VIM and ZO-1 expression via targeting FBXL10

**Fig. 7:**
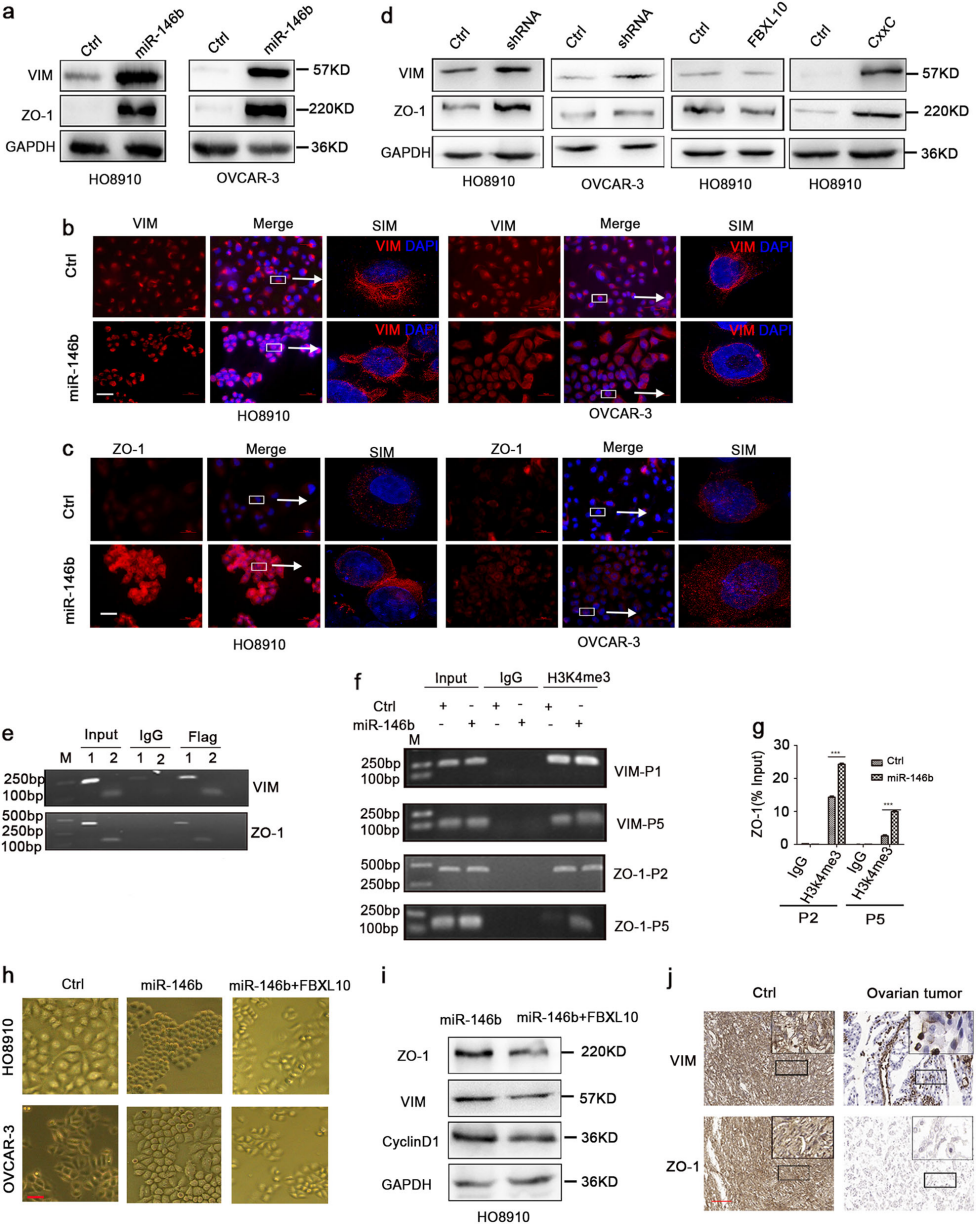
MiR-146b upregulated the expression of VIM and ZO-1 by targeting FBXL10. **a** Immunoblot analysis for VIM and ZO-1 in HO8910 and OVCAR-3 with the miR-146b overexpression. **b** Immunofluorescence staining of VIM in HO8910 and OVCAR-3 cells. Scale bars represent 50 μm. **c** Immunofluorescence staining of ZO-1 in HO8910 and OVCAR-3 cells. Cell nuclei were stained with DAPI. Scale bars represent 50 μm. **d** The expression level of VIM and ZO-1 in the FBXL10-knockdown cells and FBXL10-overexpressing cells. **e** ChIP analysis of FBXL10 binding at the VIM, ZO-1 locus in HO8910-FBXL10 cells. 1, 2 represent different promoter sites of VIM (1:-1183bp of VIM promoter,2: -153bp of VIM promoter), ZO-1 (1: -2453bp of ZO-1 promoter, -1953bp of ZO-1 promoter). **f** ChIP analysis of H3K4me3 binding at the VIM (VIM-P1: -1183bp of the of VIM promoter, VIM-P5: -153 bpof VIM promoter), ZO-1 (ZO-1-P2: -2453 bp of ZO-1 promoter, ZO-1 P5: -1953bp of ZO-1 promoter) locus in HO8910 cells transduced with miR-146b lentivirus. **g** H3K4me3 enrichment in sites of *ZO-1* promoter with the overexpression of miR-146b. **h** Representative images of the miR-146b-overexpressing cells with overexpressing FBXL10 in HO8910 and OVCAR-3 cells. **i** Immunoblot analysis of VIM, ZO-1, and Cyclin D1 of the miR-146b-overexpressing cells with FBXL10 overexpression. **j** Representative images of immunohistochemical staining for VIM and ZO-1 in normal ovary tissues and ovarian tumor tissues (control samples, *n* = 8; cancer samples, *n* = 10). Scale bars represent 100 μm. ***p < 0.001

Since targeted overexpression of miR-146b induced morphological and functional changes, which gave some clue of epithelial-to-mesenchymal transition (EMT)/mesenchymal-to-epithelial transition (MET). To determine whether these results correlated with EMT or MET, we examined the expression of mesenchymal and epithelial markers in miR-146b overexpressing cells . Notably, ovexpression of miR-146b did not undergo complete EMT or MET. Instead, the miR-146b overexpression presented both EMT and MET transcriptomic signatures (Fig. 7a and Figure S4a). Both VIM and ZO-1 were upregulated after miR-146b overexpression. Thus, we next performed an immunofluorescence assay to directly visualize the effect of miR-146b on VIM and ZO-1 localization (Fig. 7b, c and Figure S4b). Moreover, the expression of mesenchymal and epithelial markers was also negatively regulated by FBXL10 (Fig. 7d and Figure S4c), and the regulation of VIM and ZO-1 depends on its CxxC domain. To determine whether FBXL10 directly regulated *VIM* and *ZO-1* genes, we conducted chromatin immunoprecipitation (ChIP) assay on the binding of FBXL10 to their promoters. As expected, ChIP assay using an anti-Flag antibody revealed the direct binding of FBXL10 to the* VIM* and *ZO-1* promoters (Fig. 7e). Additional ChIP assay revealed a considerable increase in H3K4me3 levels at the *ZO-1* gene promoter with miR-146b overexpression (Fig. 7f, g), but no significant changes were observed in H3K4me3 enrichment at the promoter of *VIM* (data not shown). These results demonstrated that ZO-1 and VIM were direct targets of FBXL10, and suggested that FBXL10 regulated the expression of ZO-1 through H3K4me3 demethylation. We further attempted to rescue the cell phenotypes by expressing wild-type FBXL10 without 3′-UTR, and discovered that the instantaneous expression of FBXL10 in miR-146b overexpression cells almost restored the cell morphology (Fig. 7h). A western blot analysis also revealed that the expression of cyclin D1, VIM, and ZO-1 was downregulated after FBXL10 overexpression (Fig. 7i). Finally, we demonstrated that VIM and ZO-1 were highly expressed in the normal ovary tissues (Fig. 7j). These results suggested that miR-146b overexpression mediated the upregulation of Cyclin D1, VIM, and ZO-1, which might contribute to reduced invasion and increased proliferation in ovarian cancer.

### MiR-146b overexpression induced a similar phenotype of primary cancer in mouse models

**Fig. 8:**
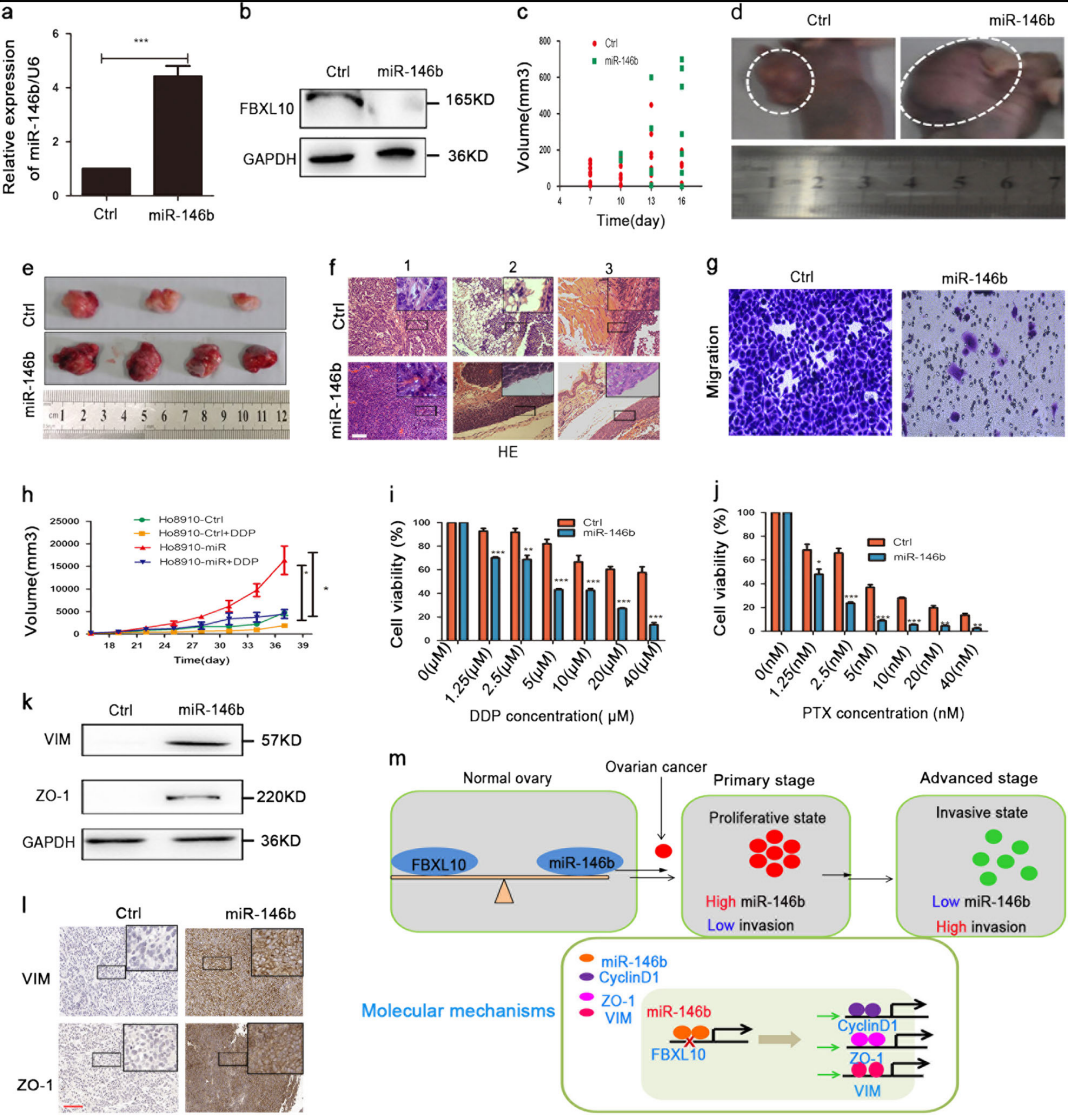
Overexpression of miR-146b enhanced xenograft tumor growth but also reduced the invasion of tumor cells. **a** qPCR analysis of miR-146b in the mouse tumors. **b** Protein level of FBXL10 in primary cultured tumor cells. **c** The time and volume of tumors appeared after transplantation. **d**, **e** The represented tumors from nude mice after miR-146b overexpression. **f** Representative H&E-stained images of mouse tumors. 1 represents the internal structure of the human xenograft tumor, 2 represents the control tumor has infiltrated into the adipose tissue of the skin, 3 represents the control tumor has infiltrated into muscle tissue of the skin.Scar bars represent 100 μm. **g** The representative images of the transwell migration assay in the miR-146b overexpression groups (200×). **h** During the tumor growth, the tumor sizes in different groups (control, *n* = 3; control + DDP, *n* = 4; miR-146b, *n* = 4; miR-146b + DDP, *n* = 4). **i**, **j** Representative images of ovarian tumor cells with different concentrations of DDP/PTX treatment. **k**, **l** Immunoblot analysis of VIM and ZO-1 in mouse models. Scar bars represent 100 μm. **m** Proposed model for epigenetic regulation of the miR-146b in ovarian cancer. In normal ovary cells, the expression between miR-146b and FBXL10 allows for a mutual balance of each partner, further ensuring the normal function of ovary. In early-phase ovarian cancer cells, high miR-146b expression promotes cell proliferation but inhibits cell migration, following this, an unknown signal reduced the levels of miR-146b, further upregulated the FBXL10 expression, and then reduced Cyclin D1, VIM, and ZO-1 expression, thus resulting in tumor metastasis and chemotherapy failure. *p < 0.05, ** *p* < 0.01, ***p < 0.001

Because miR-146b has marked effects on ovarian cancer cells in vitro, we further extend our findings in vivo. Ovarian cancer cells stably overexpressing miR-146b or control cells were injected into the nude mice. Figure 8a revealed that this lentivirus efficiently increased the expression of miR-146b in vivo. Figure 8b indicated that miR-146b downregulated FBXL10 in human xenograft tumor cells. Furthermore, tumors overexpressing miR-146b were observed later, but they grew faster than the control tumors (Fig. 8c). In addition, the control tumors were nodular, firm, and white, while the miR-146b overexpression tumors were ovoid, much soft, and deep red in appearance (Fig. 8d, e). The hematoxylin and eosin-stained (H&E) images indicated that the miR-146b-overexpressing tumors did not infiltrate any tissue of the skin (Fig. 8f). In contrast, the control tumors with low miR-146b expression had already infiltrated into the adipose tissue and muscle tissue of the skin (Figs. 8f2, 3). The transwell assay revealed that miR-146b overexpression significantly reduced cell migration (Fig. 8g). It had been reported that high proliferation without metastasis is the most prominent and well-characterized feature of primary cancer^22,23^. We think miR-146b induced a similar phenotype of primary cancer in mouse models.

We further investigated the effect of chemotherapeutic agents on ovarian cancer cells overexpressing miR-146b in vivo. Mice with miR-146b overexpression were more sensitive to DDP treatment than the control mice (Fig. 8h and Figure S5). MiR-146b overexpression also significantly improved the response of human xenograft tumor cells to DDP and PTX treatment (Fig. 8i, j). However, DDP treatment did not alter the tumor morphology (Figure S6). Further, we identified the expression of VIM and ZO-1 in these tumors (Fig. 8k, l). An integrated model for miR-146b and ovarian cancer is described in Fig. 8m.

## Discussion

In this study, we identified a miR-146b-dependent dual signature involved in regulating cell migration and proliferation. First, our findings demonstrated a promising predictive value of miR-146b regarding EOC progression and chemotherapy response. Second, we discovered that miR-146b overexpression changed cell morphology from an invasive phenotype to a proliferative phenotype, contradicting the original cancer cell proliferation/migration hypothesis^24^. Previous studies have demonstrated that proliferation and invasion are two contrasting events in some certain condition, and tumor cells can switch between these two states^25,26^. Consistent with these findings, YB-1 protein has been reported to reduce cell proliferation but promote migration in breast cancer^23^. Then, our findings also support that those highly proliferative cells exhibited high response to chemotherapeutic agents. A similar phenomenon was also reported, in that miR-141 and miR-200a promoted tumor growth but also sensitized tumors to chemotherapeutic agents in ovarian cancer^10^. Finally, our data suggest that high miR-146b might be important for the maintenance of primary ovarian cancer. A relevant study also confirmed that the expression of miRNAs was differentially regulated during multistage tumorigenesis^27^. For instance, the levels of miR-200s were shown to be higher in localized tumors and downregulated in metastases^27,28^. Thus, ovarian cancer cells with low miR-146b seem to facilitate the invasion of cancer cells.

Human miR-146b is located on chromosome 10q24.3 (miR-Base website). As a metastasis-inhibiting miRNA, miR-146b has been reported to be involved in glioma cell migration and invasion via targeting epidermal growth factor receptor^29^. Another group demonstrated that miR-146b functions as a negative regulator of nuclear factor-κB activity in breast cancer via targeting TRAF6^30^. By contrast, miR-146b acted as an oncogene in thyroid cancer through targeting SMAD4^13^. However, a relevant concern is the mechanism through which miR-146b regulates EOC. By using bioinformatics, western blot analysis, and reporter assay, we confirmed that FBXL10 was a target of miR-146b in EOC. The misregulated expression of FBXL10 has been revealed in various cancers, including lymphoblastic leukemia, human pancreatic cancer, breast cancer, and nasopharyngeal carcinoma^17,19,31,32^. However, the role of FBXL10 in ovarian cancer remains unclear. Our data revealed that FBXL10 was upregulated in EOC, and confirmed that FBXL10 enhanced cell migration but reduced proliferation in ovarian cancer cells. It has been reported that high levels FBXL10 promoted cell migration, and led to an increase in cell size^15,33^. This is congruent with our results. But the role of FBXL10 in cell proliferation is controversial in different study models^34,35^. Furemore, this study identified a possible underlying regulatory mechanism of miR-146b in regulating EOC. We found that miR-146b upregulated cyclin D1, VIM, and ZO-1 protein expression through FBXL10, and this is then followed by low  cell migration and high cell proliferation.

Cyclin D1 plays a crucial role in cell cycle regulation. It has been reported that D-type-overexpressing cyclins contracted the G1 phase, and decreased cell size. Other studies revealed that H3K4me3 induced cyclin D1 expression by binding to its promoter^36^, indicating that FBXL10 downregulated cyclin D1 possibly through H3K4me3 demethylation. VIM and ZO-1 are important mesenchymal and epithelial markers, which have an important role in cell migration. It has been demonstrated that ovarian cancer can exhibit both mesenchymal and epithelial phenotypes for cell migration and proliferation^37^. Therefore, additional research is required to fully understand the relationship between mesenchymal and epithelial markers in EOC. Our data indicate that miR-146b overexpression enhances the expression of both VIM and ZO-1 in EOC. This phenotype might inhibit cell motility to suppress dissemination from primary tumors.

## Conclusions

Together, our findings challenge previous models of EOC progression that cell proliferation and migration occur simultaneously. Our findings suggest the important role of miR-146b-FBXL10 pathway in reduced invasion and increased proliferation in EOC. The present study mainly focused on uncontrolled tumor proliferation, and this property has been exploited in the development of anti-cancer treatments, and future studies should elucidate the additional traits/mutations to trigger phenotypic changes that enhance migration.

## Materials and methods

### Patient recruitment and tissue sample collection

All ovarian samples, including control and cancer tissues, were collected from the Affiliated Hospital of Jiangsu University, the Affiliated People’s Hospital of Jiangsu University (Zhenjiang, China), and the Wuxi Maternal and Child Health Care Hospital (Wuxi, China) from 2012 to 2017. The control ovarian tissues were derived from patients who suffered from abdominal masses, uterine fibroids, and other diseases, and need to have their ovaries removed. Informed consent was obtained from all patients. Samples were collected and snap frozen in liquid nitrogen within 60 min and stored at −80 °C until analysis. The tumor samples were analyzed according to the principles expressed in relevant national law regarding the protection of biomedical research participants. This study was approved by the Medical Ethics Committee of Jiangsu University.

### Cell culture

EOC cell lines SKOV3 and HO8910 were from Prof. Xiaodong Lu, A2780 and OVCAR-3 were from Prof. Xiaoming Zhou, and human embryonic kidney cell line HEK 293T was from Prof. Wenrong Xu. SKOV3, HO8910, and HEK 293T cells were grown in the Dulbecco’s modified Eagle’s medium (DMEM) supplemented with 10% fetal bovine serum (FBS). Human ovarian cancer cells A2780 and OVCAR-3 were maintained in RPMI-1640 medium with 10% FBS. All cells were incubated at 37 °C in a humidified air atmosphere containing 5% CO_2_.

### Reagents and antibodies

miR-146b mimics and miR-146b inhibitors as well as miRNA mimic and inhibitor negative controls (Bioneer, South Korea), polybrene (abs42025397, Absin, China), puromycin (abs42025969, Absin, China), Simple-ChIP Kit (#91820, Cell Signaling Technology, USA), EdU Cell Proliferation Assay Kit (C10812-1, Ribobio, China), Annexin V-APC/7-AAD Apoptosis Kit (70-AP105-30, MultiSciences, China), and F-actin (40734ES75, Proteintech, China) were used in this study. The antibodies for western blot were as follows: anti-β-actin (1:10,000, 1sc-58673, Santa Cruz, USA), anti-cyclin D1 (1:1000, #2922, Cell Signaling Technology, USA), anti-cyclin E (1:1000, sc-247, Santa Cruz, USA), anti-cyclin B1 (1:1000, sc-245, Santa Cruz, USA), anti-cyclin A (1:1000, sc-751, Santa Cruz, USA), anti-GAPDH (1:5000, 60004-1-Ig, Proteintech, China), anti-TRAF6 (1:1000, ab33915, Abcam, USA), anti-IRAK1(1:1000, ab208009, Abcam), anti-FBXL10 (1:1000, ab5199, Abcam, USA), anti-FBXL10 (1:1000, #09864, Millipore, USA), anti-H3K36me2 (1:1000, ab9049, Abcam, USA), anti-H3 (1:1000, #4499, Cell Signaling Technology, USA), anti-H3K4me3 (1:1000, #9751, Cell Signaling Technology, USA), anti-Flag (1:1000/1:50, F1804, Sigma, USA), anti-PCNA (1:1000, #13110, Cell Signaling Technology, USA), anti-VIM (1:1000, 1:100, #5741, Cell Signaling Technology, USA), and anti-ZO-1 (1:1000, 1:100, #8193, Cell Signaling Technology, USA). The primary antibodies for the IHC were as follows: anti-FBXL10 (1:100, ab85807, Abcam, USA), anti-VIM (1:200, 10366-1-AP, Proteintech, People's Republic of China), and anti-ZO-1 (1:200, 21773-1-AP, Proteintech, People's Republic of China). The primary antibodies for IFC were anti-VIM (1:200, #5741, Cell Signaling Technology, USA), and anti-ZO-1 (1:200, #8193, Cell Signaling Technology, USA). The secondary antibodies used were as follows: Alexa Fluor 594-conjugated anti-rabbit IgG (1:200, SA00006-4, Proteintech, People's Republic of China), horseradish peroxidase (HRP)-conjugated anti-mouse (1:100,000, 70-GAM007, MultiSciences, People's Republic of China), HRP-conjugated-anti-rabbit IgG (1:100,000, 70-GAR007, MultiSciences, People's Republic of China), and peroxidase-conjugated AffiniPure rabbit anti-goat IgG (1:100, SA00001-4, Proteintech, People's Republic of China). Cell protein extraction reagent was obtained from CW BioTec (CW0889, Beijing, People's Republic of China). 4′,6-Diamidino-2-phenylindole (DAPI) was obtained from Beyotime Institute of Biotechnology (Shanghai, People's Republic of China).

### Quantitative reverse transcription-PCR

Total RNA was extracted using the Trizol reagent (Takara, Japan). Complementary DNA (cDNA) was synthesized using a reverse transcriptase (Promega), and qPCR was performed as previously described^38^. U6 small RNA for mature miRNA was amplified as an internal standard. Fold changes were calculated using the standard curve according to the manufacturer’s protocol. Each experiment was independently repeated three times. All primers used for qPCR are listed in Table 2.

### Plasmid construction

pTY-EF1a-FBXL10-Flag plasmid (#61739, Addgene, USA) and pTY-EF1a-FBXL10 (del CxxC domain)-Flag (#61740, Addgene, USA). For miR-146b overexpression, pri-miR-146b was directly cloned from human genomic DNA into pcDNA3.1 expression vector at the *Hin*dIII and *Not*I sites. The resulting construct was termed pcDNA3.1-miR-146b. The *FBXL10*-3′-UTR was cloned from ovarian cell genomic DNA into psiCHECK-2 luciferase reporter vector at the *Not*I and *Xho*I sites. The *FBXL10*-3′-UTR mutant was generated using four primers according to an overlapping PCR. For stable overexpression of miR-146b, miR-146b gene from pcDNA3.1-miR-146b vector was cloned into Psmpuw vector at the *Psh*AI site. For target gene knockdown, the shRNA targeting human FBXL10 was cloned into the PLKO.1-TRC cloning vector at the *Age*I/*Eco*RI sites. All constructs were verified through plasmid DNA sequencing. The primers for plasmid construction are listed in Table 2.

**Table 2:** Primers for PCR and Construction of Plasmids in this study

miRNA-146b-RT	GTCGTATCCAGTGCAGGGTCCGAGGTATTCGCACTGGATACGAC AGCCTA
miRNA-146b-S	GCGTGAGAACTGAATTCCA
miRNA-146b-A	GTGCAGGGTCCGAGGTAT
hsa-β-actin-F	CACGAAACTACCTTCAACTCC
hsa-β-actin-R	CATACTCCTGCTTGCTGATC
U6-F	CTCGCTTCGGCAGCACA
U6-R	AACGCTTCACGAATTTGCGT
hsa-Fbxl10-F	CAAGGAGCAGAAGATGAACCG
hsa-Fbxl10-R	TGGGGCTTCTCGTATTTCCG
hsa-pre-miR-146b-F	CCCAAGCTTCCTCAACTTACTCATCC
hsa-pre-miR-146b-R	ATTGCGGCCGCGAGCCCAAACCATC
Fbxl10-3′-UTR-F	CCGCTCGAGTCCAAGGATAAGTATGTAAAT
Fbxl10-3′-UTR-R	ATTGCGGCCGCCAACAAAGGTAAAATCG
Fbxl10-UTR-M-F	CTCTGAAGTGTGAGTTGAACCTGCAT
Fbxl10-UTR-M-R	ATGTTACAAACCTAAATGCAGGTTC
Fbxl10-shRNA-F	CCGGCCTGAGGAAGAAGCGGAAATACTCGAGTATTTCCGCTTCTTCCTCAGGTTTTTG
Fbxl10-shRNA-R	AATTCAAAAACCTGAGGAAGAAGCGGAAATACTCGAGTATTTCCGCTTCTTCCTCAGG
VIM-P1-F1	GACCATCCCTTTGTCTCG
VIM-P1-R1	CAGTTCGCATTTCCTCC
VIM-P5-F1	AAAACCTTCCCGGTGCAAT
VIM-P5-R1	TTTGCTCGAATGTGCGGACTT
ZO-1-P2-F1	GAGACAAGATGTCCGCCAGAGC
ZO-1-P2-R1	GGACCAACCACTCCTCCAAAGA
ZO-1-P5-F1	GGAAAAGTGAAAAATGTCAGTGC
ZO-1-P5-R1	AAAAGGTGGTGATGAAAGACC

### Transfection and lentiviral transduction

For miRNA mimics, miRNA inhibitors, and negative control oligos, cells were transfected with 100 nM indicated oligonucleotides using Lipofectamine 2000 (Invitrogen). For viral transduction, the lentivirus was generated through the co-transfection of expression plasmid with packaging plasmids into HEK 293T cells. The virus-containing supernatant was harvested at 48 and 72 h post transfection. Ovarian cancer cells were transduced with the lentivirus and selected with 1 μg/mL of puromycin for 2 weeks.

### Monolayer wounding assay, in vitro cell migration, and invasion assays

For the wound healing assay, cells were seeded into a 6-well plate. When cells reached 80–90% confluence, they were scratched using a 10 μL pipette tip and washed three times with phosphate-buffered saline (PBS). After 24 h, migration was assessed microscopically. Cell migration and invasion were also performed using Corning transwell insert chambers. Cells suspended in 200 μL of serum-free DMEM medium were placed into the upper chamber of the insert with or without Matrigel. After 24 h incubation, cells in the upper chamber were removed carefully and cells that had migrated through the membrane were stained with crystal violet. Five randomly selected fields were chosen for cell number quantification, and cells that had invaded through the membrane were counted under a light microscope.

### Cell proliferation, apoptosis, and drug-sensitive test

#### Cell count assay

Cells were seeded at 5 × 10^3^ cells per well in a 24-well plate and cultured for different time periods. Cells were then subjected to trypsin, and counted using a microscope. Three independent wells were counted by each group, and the average cell number was calculated.

#### EdU proliferation assay

Cells were seeded in 96-well plates. After 48 h of culture, cells were incubated with EdU for 2 h before fixation and permeabilization, and EdU stainings were performed according to the manufacturer’s protocol. The cell nuclei were stained with DAPI at a concentration of 1 µg/mL for 10 min. The proportion of cells that incorporated EdU was determined through fluorescence microscopy.

#### Cell cycle analysis

Cells were subjected to trypsin, washed twice in PBS, resuspended in 200 μL propidium iodide buffer (50 μg/mL propidium iodide, EDTA, 0.1% Triton X-100, 10 μg/mL ribonuclease A), and incubated for 0.5–1 h at 4 °C in the darkness. The DNA content of the cells was measured using an FACS.

#### Analysis of cell apoptosis

Cells were processed using the Annexin-APC/7-AAD Apoptosis Assay Kit according to the manufacturer’s protocol and 50,000 events were analyzed with a FACS flow cytometer.

#### Drug-sensitive test

Cells were seeded in a 24-well plate at 5 × 10^3^ cells per well and incubated in medium containing different concentration of cisplatin (DDP) or paclitaxel (PTX) at various concentrations for 48 h. Subsequently, the cells were treated with trypsin and counted using a microscope. Three independent wells were counted for each group.

### Western blotting

Western blotting was performed on total lysates or nuclear fractions as described previously^39^. In brief, an equal amount of protein samples was loaded and separated in 10% (8%, 12%, and 15%) sodium dodecyl sulfate-polyacrylamide gel depending on the molecular weight of the proteins and transferred to polyvinylidene fluoride membranes. Membranes were blocked with 5% bovine serum albumin (BSA) for 2 h at room temperature and then incubated in primary antibodies overnight at 4 °C, followed by incubation with secondary antibodies with HRP at room temperature for 1 h. The target proteins were subsequently visualized using enhanced chemiluminescence.

### Luciferase reporter assay

HEK 293T cells were cultured in a 24-well plate at a density of 70–90%, and then the cells were co-transfected with 400 ng pcDNA3.1 vector and 80 ng of psiCHECK-2 luciferase vector using Lipofectamine 2000 reagent (Invitrogen). Cell extracts were prepared 24 h after transfection, and the luciferase activity was measured using a Dual Luciferase Reporter Assay System (Promega).

### Immunohistochemistry

IHC staining was performed in the paraffin-embedded tissue samples. First, the slides were deparaffinized in xylene and rehydrated in a series of graded alcohol solutions. Then, 3% hydrogen peroxidase was used to block endogenous peroxidase. Second, the slides were immersed in 10 mM citric acid buffer with heat for the antigen retrieval. Then, the slides were blocked with FBS before being incubated with a primary antibody overnight, followed by HRP-conjugated secondary antibody incubation for 1 h at room temperature. Antibody binding was detected by 3,3′-diaminobenzidine (DAB), and the reaction was stopped by immersing the tissue sections in distilled water once a brown color appeared. Tissue sections were counterstained using hematoxylin, dehydrated in graded ethanols, and mounted under a light microscope.

### Immunofluorescence staining and F-actin cytoskeleton staining

DAPI was used for staining of the nuclei. For immunofluorescence staining, the culture medium was aspirated, and the cells were washed three times with cold PBS. Then, the cells were fixed in 4% paraformaldehyde and permeabilized using 0.3% Triton X-100. Subsequent cells were blocked with 3% BSA and incubated with antibodies against VIM and ZO-1. After washing in PBS three times, cells were incubated with secondary antibodies conjugated with Alexa Fluor 594. Finally, the cells were mounted on a fluorescence microscope (Axio Observer A1, Zeiss, Germany) or a DeltaVision OMX Blaze™ System (Applied Precision GE Healthcare, USA). For F-actin cytoskeleton staining, cells were fixed in 4% formaldehyde and stained with rhodamine-conjugated phalloidin for 30 min in darkness, and cells were additionally stained with DAPI in darkness. Then, the cells were analyzed under a Leica TCS SP5 II inverted confocal laser scanning microscope (Leica, Germany) and a Nikon N-STORM (Nikon, Japan) microscope.

### ChIP assay

ChIP assays were performed according to the manufacturer’s recommendations. Briefly, cells were cross-linked with 1% formaldehyde for 10 min. Cross-linked cells were prepared and chromatin was digested with micrococcal nuclease, and then the nuclear membrane was broken by ultrasonic wave. Following overnight incubation with specific antibodies, 30 μL of protein G magnetic beads were added at 4 °C for 2 h. The immunoprecipitates were serially washed to remove nonspecific binding. After reverse cross-linking, the DNA samples were purified and analyzed by PCR. The primer sequences for the VIM promoter and ZO-1 promoter are listed in Table 2. The final results represent the percentage of input chromatin, and error bars indicate the standard deviations from the triplicate experiments.

### Tumor xenograft experiments

All animal experiments in this study were complied with the guidelines of the Animal Research Ethics Board of Jiangsu University. HO8910 cells were infected with a control lentivirus (HO8910/control) or a miR-146b lentivirus (HO8910/miR-146b), and were injected subcutaneously into the left side of each mouse (female nude mice, 6–8 weeks old, obtained from the Animal Center of Yangzhou University, Yangzhou, People's Republic of China). The mice were checked daily, and tumor diameters were measured every 3 days. Tumor volume was quantified as *V* = (length × width^2^)/2. After 16 days, the mice were randomly divided into four groups: HO8910/control, HO8910/control + DDP, HO8910/miR-146b, and HO8910/miR-146b + DDP. For chemotherapy treatment, DDP was administered at a dose of 3 mg/kg every 3 days, physiological saline was set as the negative control. Four weeks after treatment, all animals were sacrificed, and part of the xenografts was immediately processed for primary cell culture, and the others were harvested for subsequent experiments. Tumor tissues were fixed in 10% formalin and embedded in paraffin routinely.

### Statistical analysis

Data were expressed as the mean ± SD from at least three independent experiments. Statistical comparisons of the results between groups were performed using the Student’s *t* test, one-way analysis of variance, and two-way analysis of variance. *P* < 0.05 was considered statistically significant.

## Electronic supplementary material

supplementary figure legends

Figure S1

Figure S2

Figure S3

Figure S4

Figure S5

Figure S6
