# Supplementary material for: miR-146b promotes cell proliferation and increases chemosensitivity, but attenuates cell migration and invasion via FBXL10 in ovarian cancer
Source: Cell Death Dis. 2018 Nov 8;9(11):1123. doi: 10.1038/s41419-018-1093-9 (PMC6224598; doi:10.1038/s41419-018-1093-9)
Supplement: Supplementary file 1 — supplementary figure legends [file 41419_2018_1093_MOESM1_ESM.docx]

**Supplementary figure legends**

**Figure S1.** **a.** Representative images of ovarian cancer cells transduced with miR-146b or control lentrivirus using a fluorescence microscope. Scale bars represent 200 μm. **b.** The expression level of miR-146b in ovarian cancer cells transduced with miR-146b lentrivirus was measured by qPCR. **c.** The cell size of ovarian cancer cells with miR-146b overexpression using FACs analysis. **d.** Detection of TRAF6 and IRAK1 protein expression of ovarian cancer cells by Western blot. **e.** Representative flow cytometry of apoptosis of HO8910 and OVCAR-3 cells which were stained with Annexin V and 7-AAD. *p< 0.05, ** p<0.01, ***p< 0.001.

**Figure S2.** Overexpression of miR-146b promoted HO8910 cells growth. 5000 cells were seeded into a 24-well culture plate. After 2 days incubation, the representative images of HO8910 cells tranduced with miR-146b lentrivirus were shown.

**Figure S3. a.** Schematic representation of the miR-146b and its targeting sites in the 3’UTR of ELAVL1 and Lin28a, indicating the binding sites and the corresponding mutations. **b.** The luciferase activity change after co-transfected with the luciferase reporter vector and pcDNA3.1-pri-miR-146b or control structures into 293T cells**. c.** In vitro transwell migration assay of HO8910 and SKOV3 cells after transfected with miR-146b mimic and inhibitor. *p< 0.05, ** p<0.01, ***p< 0.001.

**Figure S4. a.** Detection of E- cadherin and N- cadherin protein expression of ovarian cancer cells with miR-146b overexpression by Western blot. **b.** Immunofluorescence staining of VIM in HO8910 and OVCAR-3 cells. **c.** Detection of E- cadherin and N- cadherin protein expression after FBXL10 knockdown by Western blot.

**Figure S5.** The representative tumors from nude mice after DDP treatment.

**Figure S6.** Representative H&E-stained images after DDP treatment. Scale bars represent 100 μm.
