# Supplementary figures and images for: miR-146b promotes cell proliferation and increases chemosensitivity, but attenuates cell migration and invasion via FBXL10 in ovarian cancer
Source: Cell Death Dis. 2018 Nov 8;9(11):1123. doi: 10.1038/s41419-018-1093-9 (PMC6224598; doi:10.1038/s41419-018-1093-9)

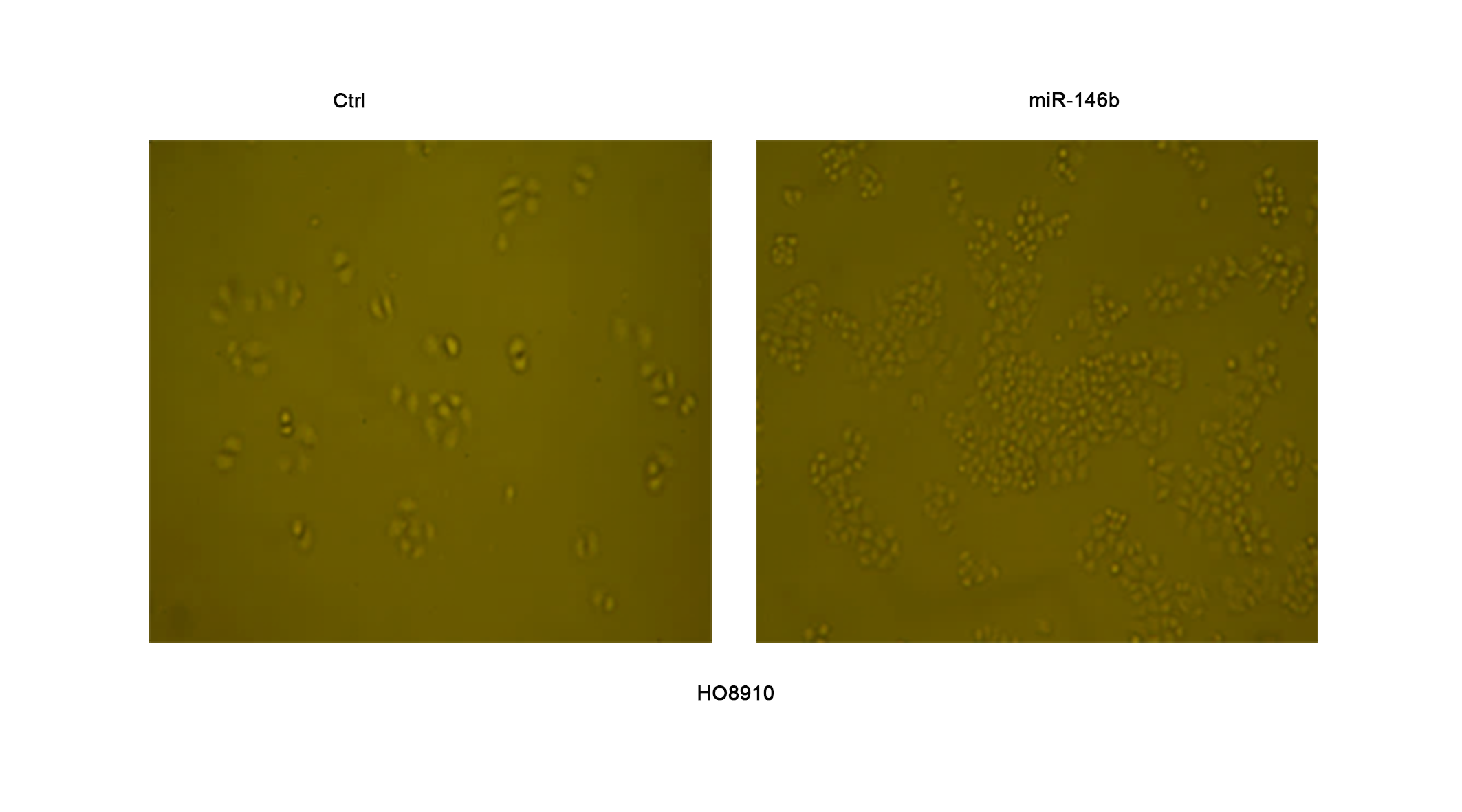

Supplement: Supplementary file 3 — Figure S2 [file 41419_2018_1093_MOESM3_ESM.tif]
